# Mus81 knockdown improves chemosensitivity of hepatocellular carcinoma cells by inducing S‐phase arrest and promoting apoptosis through CHK1 pathway

**DOI:** 10.1002/cam4.588

**Published:** 2015-12-29

**Authors:** Fan Wu, Wei‐Jia Chen, Lun Yan, Guo‐Qian Tan, Wei‐Tao Li, Xuan‐Jin Zhu, Xiao‐Chuan Ge, Jian‐Wei Liu, Bai‐Lin Wang

**Affiliations:** ^1^ Department of Hepatobiliary SurgeryGuangzhou Red Cross Hospital /Fourth Affiliated Hospital of Jinan UniversityTongfu Roud 396Guangzhou510220China

**Keywords:** Apoptosis, cell cycle arrest, chemosensitivity, CHK1, hepatocellular carcinoma, Mus81

## Abstract

As a critical endonuclease in DNA repair, Mus81 is traditionally regarded as a tumor suppressor, but recently correlated with the sensitivity of mitomycin C and 5‐fluorouracil in colon cancer and breast cancer cells. However, its role in chemosensitivity of other human malignancies still remains unknown. This study therefore aims to investigate the effects of Mus81 knockdown on the chemosensitivity of hepatocellular carcinoma (HCC), a usually chemorefractory tumor, and explore the underlying mechanisms. Mus81 expression in HepG2 and Bel‐7402 HCC cell lines was depleted by lentivirus‐mediated short hairpin RNA and the elevated sensitivity of these Mus81‐inhibited HCC cells to therapeutic agents, especially to epirubicin (EPI), was evidenced by MTT assay and an HCC chemotherapy mouse model. Flow cytometric analysis also showed that Mus81 knockdown lead to an obvious S‐phase arrest and an elevated apoptosis in EPI‐treated HepG2 and Bel‐7402 cells, which could be rescued by CHK1 inhibition. The activation of CHK1/CDC25A/CDK2 pathway was also demonstrated in Mus81‐inhibited HepG2 cells and xenograft mouse tumors under EPI treatment. Meanwhile, the apoptosis of HepG2 cells in response to EPI was remarkably promoted by Mus81 knockdown through activating p53/Bax/Caspase‐3 pathway under the controlling of CHK1. In addition, CHK2 inhibition slightly raised CHK1 activity, thereby enhancing the S‐phase arrest and apoptosis induced by EPI in Mus81‐suppressed HCC cells. In conclusion, Mus81 knockdown improves the chemosensitivity of HCC cells by inducing S‐phase arrest and promoting apoptosis through CHK1 pathway, suggesting Mus81 as a novel therapeutic target for HCC.

## Introduction

Hepatocellular carcinoma (HCC) is the fifth most common cancer in the world and the third leading cause of cancer‐related mortality 1. The incidence rates of this disease are still increasing in many countries even including the United States 1, 2. Resection and liver transplantation are the potentially curative treatments for HCC, but majority of HCC patients are diagnosed at advanced stage and have lost the opportunity to surgical treatment 3, 4. Chemotherapy is therefore an important option for those patients with advanced HCC, however, the primary or acquired chemoresistance limits the antitumor effects of chemotherapy agents, whatever single or combination 5. As numerous studies have shown, the expression of DNA repair enzymes is one of the major mechanisms that contributes to chemoresistance of HCC 6, and targeting these enzymes may shed light on developing a novel therapeutic strategies for HCC 4, 7.

Methyl methansulfonate and ultraviolet‐sensitive gene clone 81 (Mus81) is a highly conserved gene across the species and encodes a structure‐specific DNA endonuclease, which resolves holliday junctions (HJs) through constituting a heterodimer with Eme1/Mms4 and plays a critical role in the repair of double‐strand breaks (DSBs) of DNA and maintenance of chromosomal integrity 8, 9. McPherson et al. 10 have showed that 73% of Mus81^−/−^ mice and 50% of Mus81^+/−^ mice died of various spontaneous tumors such as lymphoma, breast cancer, and prostate cancer, therefore Mus81 was traditionally regarded as a potent tumor suppressor. Our previous studies also demonstrated that the downregulation of Mus81 in HCC, colorectal cancer, and gastric cancer was closely correlated with the progression and prognosis of these malignancies 11, 12, 13, 14. However, inhibition of Mus81 has recently come to attention as a novel approach to promote the chemosensitivity of human cancer cells.

Mus81‐deficient embryonic stem cells and mice are hypersensitive to DNA cross‐linking agents such as mitomycin C (MMC) and cisplatin, which are also the common agents for HCC chemotherapy 15. It is subsequently evidenced that the disruption of Mus81 gene could increase the sensitivity of MMC and cisplatin in human colon cancer cell line HCT116, and this hypersensitivity could be rescued after expressing Mus81 again, obviously indicating the involvement of Mus81 in chemosensitivity of human maligancies 16. In addition, Mus81 inhibition by small interfering RNA (siRNA) also exhibited the ability to enhance the sensitivity of 5‐fluorouracil (5‐FU) in two breast carcinoma cell lines 17, further suggesting Mus81 as a candidate therapeutic target for chemosensitization. Although Mus81 expression has also been correlated with the chemosensitivity of other human malignancies such as lung cancer 18, the role of Mus81 in the regulation of chemosensitivity in HCC still remains unclear.

Therefore, this study examined the effects of Mus81 inhibition by lentivirus‐mediated shRNA on chemosensitivity of HCC in vitro and in vivo, and also explored the underlying molecular mechanisms.

## Materials and Methods

### Cell culture and reagents

HepG2 and Bel‐7402HCC cell lines were purchased from the American Type Culture Collection and all cultured in high‐glucose DMEM containing 10% fetal bovine serum at 37°C in a humidified incubator (SANYO, Osaka, Japan) with 5% CO_2_. Epirubicin (EPI) and 5‐FU were purchased from Merck Millipore (Billerica, MA). MMC was purchased from Kyowa Hakko Kirin (Tokyo, Japan). Cisplatin was purchased from Sigma (St Louis, MO). The primary antibody against Mus81, CHK1, CDC25A, CDK2, CHK2, CDC25C, CDC2, p53, Bax, and Bcl‐2 were purchased from Abcam (Cambridge, UK). The primary antibody against phosphorylated CHK1 (Ser317), phosphorylated CHK2 (Thr68), phosphorylated CDC25C (Ser216), phosphorylated CDK2 (Tyr15), phosphorylated CDC2 (Tyr15), and phosphorylated p53 (Ser15) were purchased from Cell Signaling Technology (Danvers, MA). CHK1 inhibitor (CAS: 405168‐58‐3) was purchased from Santa Cruz Biotechnology (Santa Cruz, CA). CHK2 inhibitor (CAS: 516480‐79‐8) was purchased from Merck Millipore. Caspase‐3 activity assay kit was purchased from Chemicon International (Temecula, CA).

### Lentivirus‐mediated short hairpin RNA

The Lentivirus‐mediated short hairpin RNA (shRNA) expressing vector GV248 was purchased from Genechem Technology (Shanghai, China). The sequences of shRNA‐targeting Mus81 (selected from five putative candidate sequences, data not shown) were: sence: 5′‐CCGGGAGTTGGTAC TGGATCACATTCTCGAGAATGTGATCCAGTACCAACTCTTTTTG‐3′; antisence: 5′‐AATTCAAAAAGAGTTGGTACTGGATCACATTCTCGAGAATGTGATCCAGTACCAACTC‐3′. And the negative control shRNA sequences were: sence: 5′‐CCGGTTCTCCGAACGTGTCACGTTTCAAGAGAACGTGACACGTTCGGAGAATTTTTG‐3′; antisence: 5′‐AATTCAAAAATTCTCCGAACGTGTCACGTAAGTTCTCTACGTGACACGTTCGGAGAA‐3′. The lentiviral packaging cells, 293T cells, were infected with recombinant lentiviral expression plasmid GV248 and packaging plasmid pGC‐LV, pHelper 1.0 and pHelper 2.0 at 70% confluence with the use of Lipofectamine 2000 transfection reagent (Invitrogen, Carlsbad, CA) to produce lentivirus. Media containing lentivirus was added to the HCC cells supplied with polybrene (8 *μ*g/mL) for 4 h. After 12 h, original medium was replaced with fresh medium and lentivirus was added again. The HepG2 and Bel‐7402 cells infected with lentivirus‐targeting Mus81 were named as HepG2^shMus81^ and Bel‐7402^shMus81^, respectively, and the HepG2 and Bel‐7402 cells infected with negative control lentivirus were named as HepG2^shCtrl^ and Bel‐7402^shCtrl^, respectively.

### Western blot

Total protein was extracted and separated by SDS‐PAGE, and then transferred onto PVDF membrane (Merck Millipore). The blotted membranes were incubated with primary antibody against Mus81, phosphorylated CHK1 (Ser317), phosphorylated CHK2 (Thr68), CDC25A, phosphorylated CDC25C (Ser216), phosphorylated CDK2 (Tyr15), phosphorylated CDC2 (Tyr15), and phosphorylated p53 (Ser15), and then the corresponding secondary antibody in order. GAPDH protein was also determined by using the specific antibody (Sigma) as a loading control.

### MTT assay

HepG2^shMus81^, HepG2^shCtrl^, Bel‐7402^shMus81^, and Bel‐7402^shCtrl^ cells were plated in 96‐well plates at a density of 2 × 10^3^ cells/well, and the plates were incubated for 24 h to allow for cell attachment. Then, HepG2^shMus81^ and HepG2^shCtrl^ cells were treated with different concentrations of chemotherapeutic drugs include EPI (0.1, 0.2, 0.4, 0.8, and 1.6 *μ*g/mL), 5‐FU (1.0, 2.0, 4.0, 8.0, and 16 *μ*g/mL), MMC (0.318, 0.625, 1.25, 2.5, and 5 *μ*g/mL), and cisplatin (1.5, 3.0, 6.0, 12 and 24 *μ*g/mL) for 48 h. HepG2 cells were treated with phosphate‐buffered saline (PBS) instead of chemotherapeutic drug as control. After treatment, culture medium was removed and HepG2 cells were washed by PBS for three times, then 10 *μ*L 3‐(4,5‐dimethylthiazol‐2‐yl)‐2,5‐diphe‐nyltetrazolium bromide (MTT, 5 mg/mL) was added to each well with fresh culture medium and incubated for 4 h at 37°C in the dark. After removing the supernatant, formazan crystals formed were dissolved in 100 *μ*L dimethyl sulfoxide (DMSO) and the absorbance was measured at 490 nm by a microplate reader (Biotek, Winooski, VT). Data were analyzed from three independent experiments and then normalized to the absorbance of the wells containing double diluted H_2_O only (0%) and untreated HepG2 or Bel‐7402 cells (100%). IC50 value was taken as the concentration that caused 50% inhibition of cell viabilities and calculated by Graphpad Prism 5.0 software (Graphpad Software, La Jolla, CA). Subsequently, the reverse index (RI) was calculated using the formula: RI = IC50 in HepG2^shCtrl^ or Bel‐7402^shCtrl^ cells/IC50 in HepG2^shMus81^ or Bel‐7402^shMus81^ cells.

### Flow cytometric analysis

Cell apoptosis rate was analyzed by annexin V‐staining using the annexin V apoptosis detection kit (eBioscience, San Diego, CA) according to the manufacturer's instructions. Briefly, 1 × 10^5^ cells were stained by 5 *μ*L annexin V‐APC in the dark for 15 min and then subjected to flow cytometric analysis using a FACS Calibur flow cytometer (BD Biosciences, Franklin Lakes, NJ). To analyze the cell cycle distribution, the cells were collected and fixed in cold 70% ethanol overnight at 4°C. After washing twice with phosphate‐buffered saline (PBS), the cells were subsequently stained with propidium iodide (PI, 50 *μ*g/mL) and RNase A (100 *μ*g/mL) for 1 h and subjected to flow cytometric analysis.

### Immunofluorescence assay

HepG2^shCtrl^ or HepG2^shMus81^ cells were cultured overnight to reach 80–90% confluence on coverslips in culture dishes. All these cells were treated with EPI at a dose of 0.2 *μ*g/mL. The treated cells were further cultured in complete medium for 6, 24, or 48 h, then fixed in 4% paraformaldehyde for 10 min. The cells were permeabilized in PBS containing 0.2% Triton‐X100 for 5 min, washed, and blocked with PBS containing 5% bovine serum albumin (BSA) and 10% goat serum (Invitrogen) for 30 min. Then, the cells were incubated with antibody against Mus81, phosphorylated CHK1 (Ser317), phosphorylated CHK2 (Tyr15), or phosphorylated p53 (Ser15) overnight at 4°C. After washing, the cells were labeled with Alexa fluor 488‐conjugated secondary antibody (Invitrogen), followed by examining under a fluorescence microscope (Nikon, Tokyo, Japan). Those HepG2^shCtrl^ or HepG2^shMus81^ cells without EPI treatment were detected in the same protocol as control.

### Treatment of EPI, CHK1 inhibitor, and CHK2 inhibitor for HCC cells

To analyze the function of Mus81 in the response of HepG2 and Bel‐7402 cells to therapeutic drug, 0.7 *μ*g/mL EPI (approximate IC50 of EPI for HepG2^shCtrl^) or 1.5 *μ*g/mL EPI (approximate IC50 of EPI for Bel‐7402^shCtrl^) was added to the medium of HepG2 and Bel‐7402 cells, respectively, and maintained for 48 h. To explore the role of CHK1 or CHK2 in the chemotherapy response of Mus81‐depleted HCC cells, HepG2 and Bel‐7402 cells were treated with 0.6 nmol/L CHK1 inhibitor or 30 nmol/L CHK2 inhibitor, respectively, for 1 h and subsequently treated with EPI in the doses mentioned above.

### HCC chemotherapy mouse model

Six to eight weeks old nude mice were obtained from the Tongji University Experimental Animal Center (Shanghai, China). HepG2^shMus81^, HepG2^shCtrl^, or untreated HepG2 cells were inoculated subcutaneously on the right upper flank regions of these mice (1 × 10^7^ cells/mouse). When the diameter of xenograft tumors reached 1 cm, EPI (2 mg/kg) or 5‐FU (10 mg/kg) were injected intraperitoneally into the mice bearing tumors generated from HepG2^shMus81^ or HepG2^shCtrl^ cells every other day for 14 days. PBS was also injected intraperitoneally into the mice bearing tumors generated from untreated HepG2 cells as control. The body weights and the xenograft tumor growth of these mice were also measured every other day, and the tumor volumes (*V*) were calculated using the formula: *V *= *a *× *b* ^2^/2, where *a* and *b* are the largest and smallest tumor diameter, respectively. All the mice were killed in 24 h after the final injection, and the xenograft tumors treated with EPI were dissected out, weighed up and made into 4 *μ*m thick sections for hematoxylin and eosin (H&E) staining, immunohistochemistry and terminal deoxynucleotidyl‐transferase‐mediated d‐UTP‐ biotin nick end‐labeling (TUNEL) assay. Tumor inhibition rate was calculated as ([average tumor weight of HepG2 group ‐ average tumor weight of HepG2^shCtrl^ or HepG2^shMus81 ^group]/average tumor weight of HepG2 group) × 100% 19. Animal use and care followed institutional guidelines established by the Guangzhou Red Cross Hospital Institutional Animal Care and Use Committee.

### Immunohistochemistry and TUNEL assay

The expression of Mus81, phosphorylated CHK1 (Ser317), CDC25A, and phosphorylated CDK2 (Tyr15) in xenograft tumors was detected by immuno histochemistry (IHC) assay. Briefly, the sections of xenograft tumors were deparaffinized, rehydrated, and incubated with 3% H_2_O_2_ to block the endogenous peroxidases. Subsequently, the sections were subjected to microwave heat‐induced antigen retrieval in citrate buffer (0.01 mol/L, pH 6.0) at high power for two times, each for 7 min. After rinsing with PBS, the sections were incubated with primary antibodies, the corresponding HRP‐conjugated second antibodies in order. All sections were visualized by applying 3,3‐diaminobenzidine tetrahydrochloride (DAB) and then counterstained with hematoxylin. Apoptosis in xenograft tumors was detected by TUNEL assay using a TUNEL assay kit (Roche, Basel, Switzerland) according to the manufacturer's instructions. The results of immunohistochemistry and TUNEL assay were all observed and imaged by a light microscope (Nikon).

### Statistic analyses

Data are presented as mean ± SEM for three independent experiments. The differences between the groups were examined by using two‐tailed nonpaired Student's *t* test with Graphpad Prism 5.0 software (Graphpad Software) and *P* < 0.05 was considered as significant.

## Results

### Mus81 knockdown by lentivirus‐mediated shRNA

**Figure 1:**
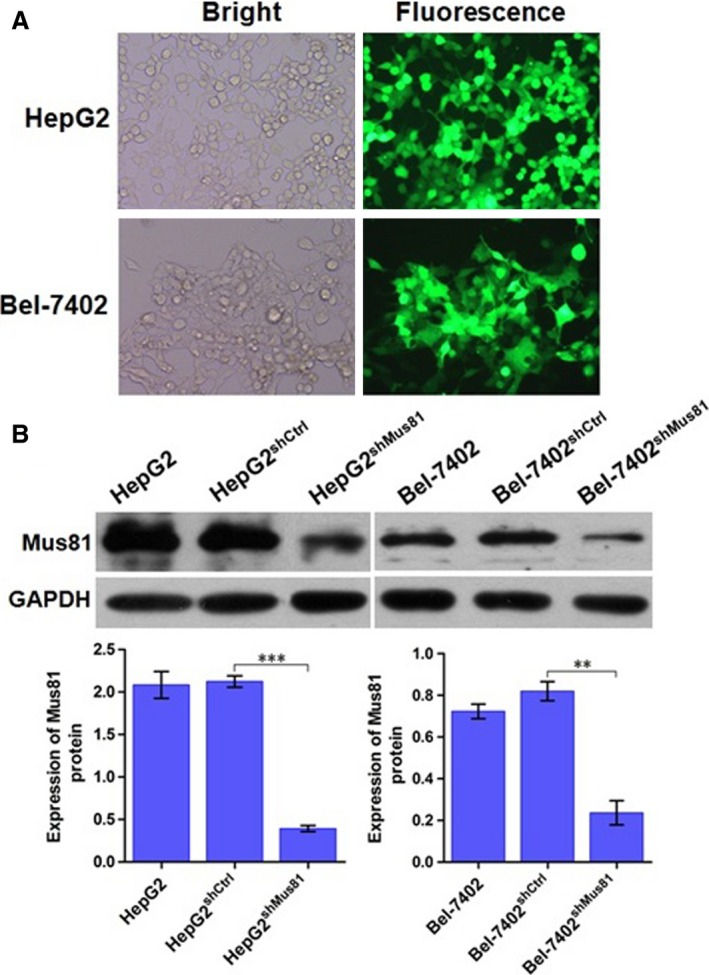
The infection efficiency and inhibition rate of Mus81 knockdown by lentivirus‐ mediated shRNA in human hepatocellular carcinoma HepG2 and Bel‐7402 cells. (A) HepG2 and Bel‐7402 cells were infected by lentivirus containing Mus81 shRNA sequence and the infection efficiencies, observed by a fluorescence microscope, in these two cells were all more than 80%. Bright, representive images in bright field. Fluorescence, the corresponding fluorescence images. (B) Western blot analysis measured the inhibition rate of Mus81 protein in HepG2 and Bel‐7402 cells after lentivirus infection. GAPDH was used as loading control. Data were presented as mean ± SEM. **P < 0.01; ***P < 0.001.

HepG2 and Bel‐7402 cells were infected with lentivirus targeting Mus81 gene. The infection efficiencies in HepG2 and Bel‐7402 cells, which were observed by a fluorescence microscope (Nikon), were all more than 80% (Fig. 1A). Seventy‐two hours after lentivirus infection, the inhibition rate of Mus81 protein was measured by western blot. Compared with the corresponding negative control cells, expression levels of Mus81 protein in HepG2^shMus81^ and Bel‐7402^shMus81^ cells were remarkablely reduced to 18.53 and 28.87% (Fig. 1B), respectively, showing the inhibition rate of Mus81 protein by lentivirus‐mediated shRNA in HepG2 and Bel‐7402 cells were 81.47 and 71.13%.

### Mus81 knockdown sensitizes HCC cells to chemotherapeutic drugs in vitro

**Figure 2:**
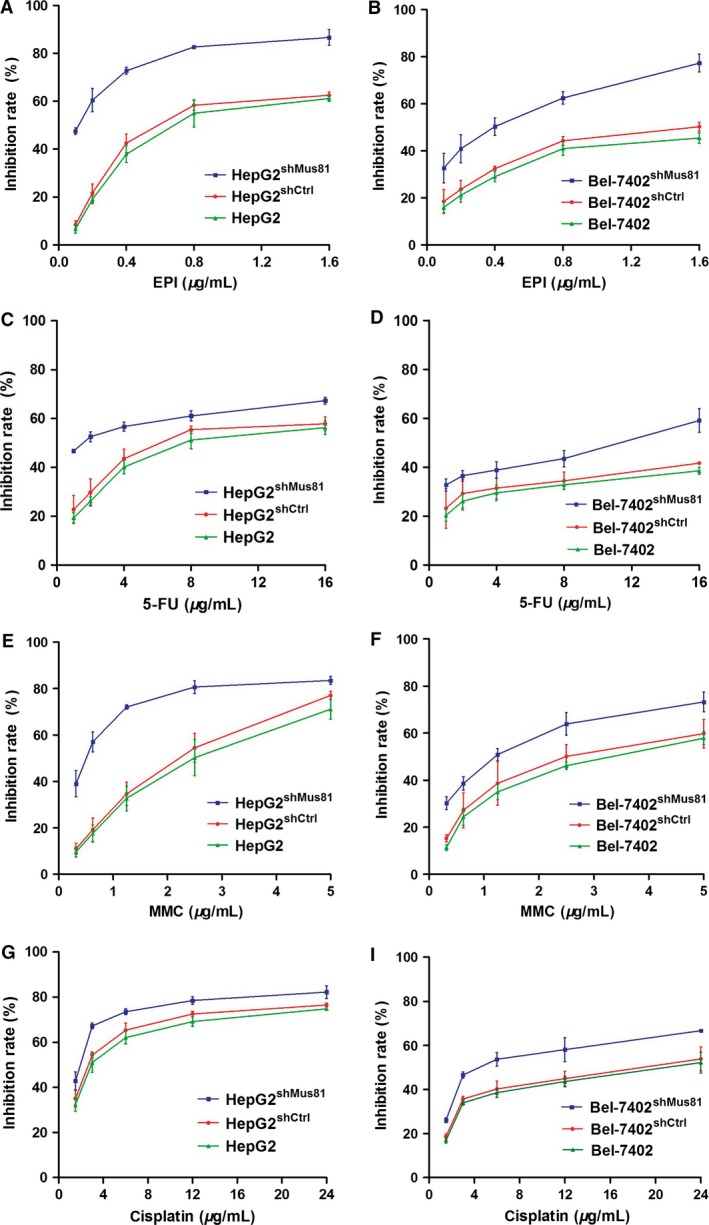
Dose‐inhibition rate curves for epirubicin (EPI), 5‐fluorouracil (5‐FU), Mitomycin C (MMC), and cisplatin in human hepatocellular carcinoma HepG2 and Bel‐7402 cells.

To determine the impact of Mus81 knockdown on the chemotherapy sensitivity of HCC cells, MTT assay was carried out and the results were analyzed by GraphPad Prism 5.0 software to establish the dose‐inhibition efficiency curves and calculate the IC50 of EPI, 5‐FU, MMC, and cisplatin to different HCC cells. As showed in Figure 2, the inhibition efficiencies of EPI, 5‐FU, MMC, and cisplatin (at every concentration we tested) to Mus81‐depleted cells were significantly higher than those negative control cells (*P* < 0.05), respectively. The IC50 values of EPI, 5‐FU, MMC, and cisplatin in HepG2^shMus81^ and Bel‐7402^shMus81^ cells were significantly decreased than HepG2^shCtrl^ or Bel‐7402^shCtrl^ cells (*P *< 0.05), and the reverse index (RI) value of EPI was the highest among the four chemotherapeutic drugs (Table 1), indicating that Mus81 knockdown could obviously enhance the chemosensitivity of HCC cells to common chemotherapeutic drugs, especially to EPI.

**Table 1:** The IC50 and RI values of chemotherapeutic drugs in HCC cells

Drugs	IC50 for HepG2 cells		RI	IC50 for Bel‐7402 cells		RI
	HepG2^shCtrl^	HepG2^shMus81^		Bel‐7402^shCtrl^	Bel‐7402^shMus81^	
EPI	0.69	0.11	6.27	1.95	0.34	5.74
5‐FU	7.24	1.54	4.70	50.11	11.90	4.21
MMC	2.04	0.48	4.25	2.61	1.15	2.27
Cisplatin	2.85	1.63	1.75	16.17	5.83	2.77

RI, reverse index; EPI, epirubicin; 5‐FU, 5‐fluorouracil; MMC, mitomycin C.

### Mus81 knockdown improves the chemosensitivity of HCC cells in vivo

**Figure 3:**
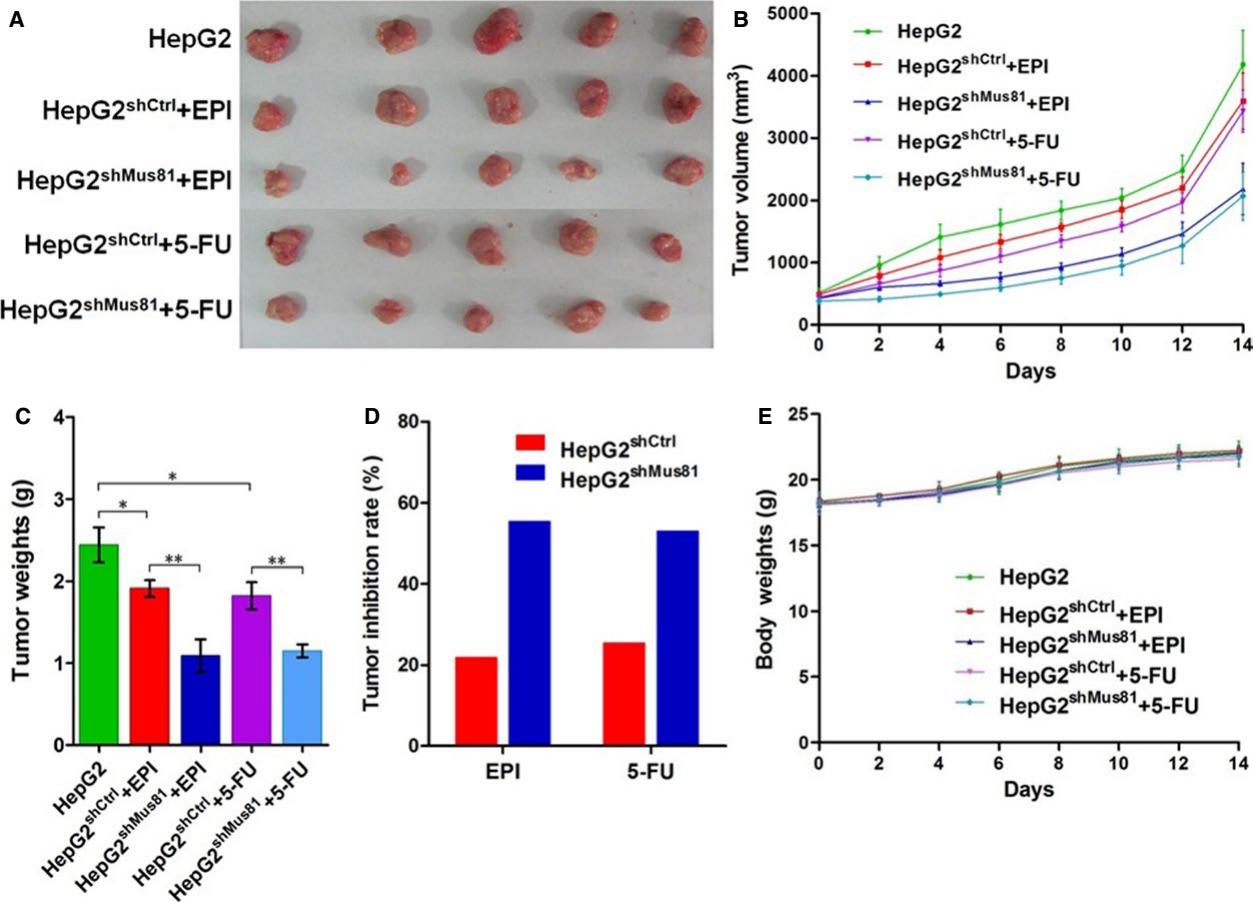
Mus81 knockdown sensitizes human hepatocellular carcinoma HepG2 cells to chemotherapeutic agents in vivo. Mice bearing tumors generated from HepG2^shMus81^ or HepG2^shCtrl^ cells were intraperitoneally injected with EPI (2 mg/kg) or 5‐FU (10 mg/kg) every other day for 14 days. PBS was also injected intraperitoneally into the mice bearing tumors came from HepG2 cells without lentivirus transfection as control. (A) Images for the resected tumors from the mice. (B) Tumor volume was measured by every other day for 14 days. (C) Tumor mass were weighed after resection. (D) Tumor inhibition rate was calculated on the base of tumor weight. (E) Mice body weight was recorded by every other day for 14 days as a surrogate marker for toxicity of EPI or 5‐FU. Data were presented as mean ± SEM,* n* = 5. *P < 0.05; **P < 0.01.

To further evaluate the implication of Mus81 knockdown on HCC chemosensitivity in vivo, an HCC chemotherapy mouse model was therefore established by combining subcutaneously injection of HCC cells (HepG2, HepG2^shCtrl^, and HepG2^shMus81^ cells) with subsequent intraperitoneally injection of chemotherapeutic drugs (EPI and 5‐FU). It was showed by this model that the growth of xenograft tumor came from HepG2^shMus81^ cells was significantly inhibited than those generated from HepG2^shCtrl^ cells during the 14‐day experimental period whatever under EPI or 5‐FU treatment (Fig. 3A and B). The tumor weights of HepG2^shMus81^ groups also decreased significantly when comparing with those of HepG2^shCtrl^ groups, and the tumor inhibition rate of EPI and 5‐FU in HepG2^shMus81^ group increased by 1.54 times (55.36% vs. 21.79%) and 1.09 times (52.99% vs. 25.39%) than those in HepG2^shCtrl^ group, respectively (Fig. 3C and D). Meanwhile, there was no significant difference in mouse body weights between different groups during the whole experiment period (Fig. 3E). All these data indicated that Mus81 knockdown could significantly improve the chemosensitivity of HCC cells in vivo without significant side effect.

### Mus81 knockdown induces S‐phase arrest in HCC cells under EPI treatment

**Figure 4:**
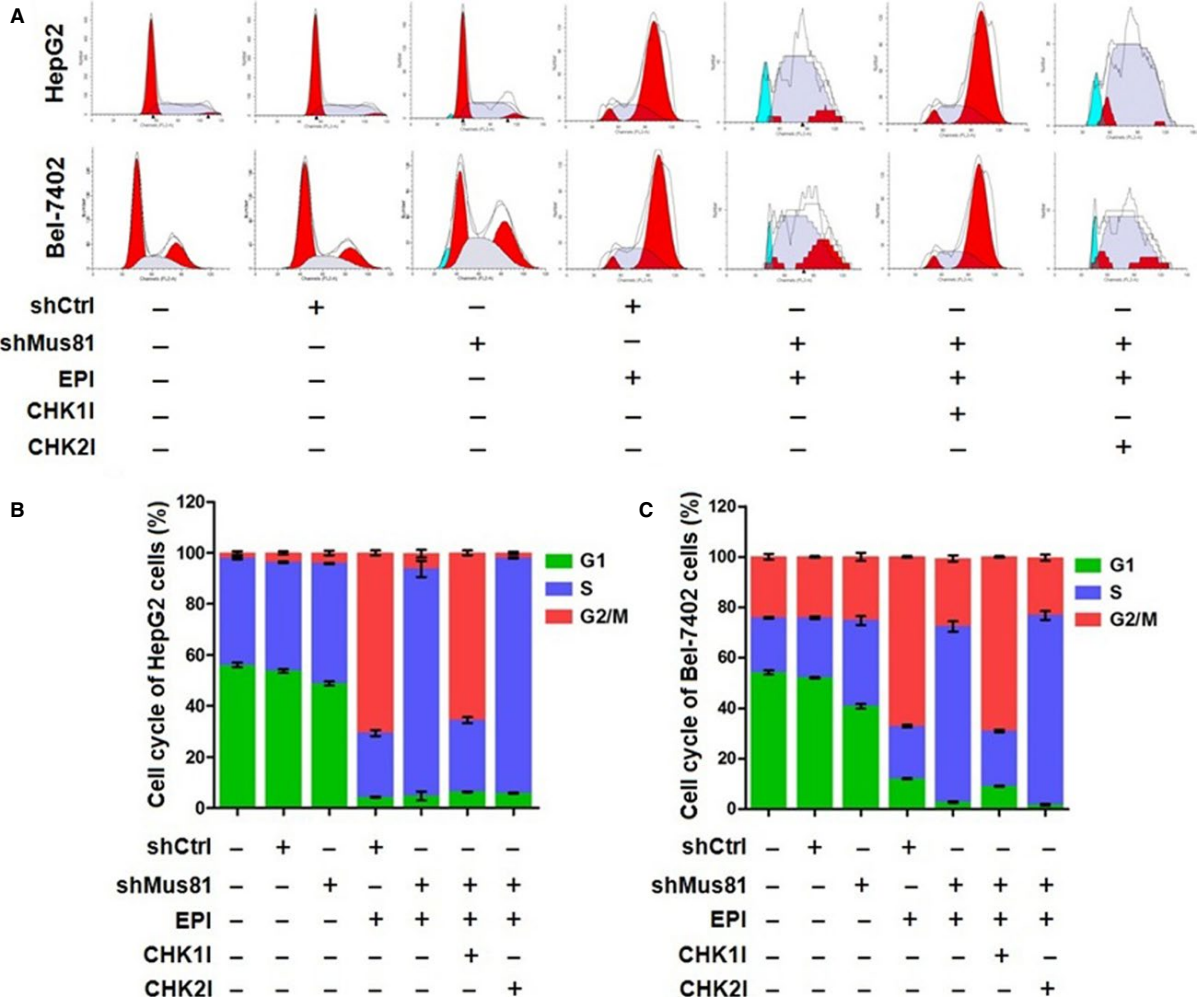
Mus81 knockdown affects cell cycle of human hepatocellular carcinoma HepG2 and Bel‐7402 cells under the treatment of epirubicin (EPI). (A) representative results of flow cytometric analysis. (B), cell cycle distribution of HepG2 cells. (C), cell cycle distribution of Bel‐7402 cells. And data are presented as Mean ± SEM. CHK1I, CHK1 inhibitor; CHK2I, CHK2 inhibitor.

Since Mus81 knockdown could inhibit the proliferation of HCC cells under chemotherapeutic drug treatment in vitro and in vivo, we asked whether this anticancer ability was induced by cell cycle arrest. Therefore, the cell cycle populations of HepG2 and Bel‐7402 cells with or without chemotherapeutic drugs treatment were determined by PI staining and flow cytometry. As shown in Figure 4, the percentages of HepG2^shMus81^ and Bel‐7402^shMus81^ cells at S phase were increased moderately from 42.63 to 47.20% or from 23.84 to 33.96% comparing with HepG2^shCtrl^ and Bel‐7402^shCtrl^ cells, and the percentages at G1 phase were also decreased significantly from 53.68 to 48.70% or from 52.06 to 40.74%, suggesting that Mus81 knockdown lead to a moderate S‐phase arrest of HCC cells. After EPI treatment, the percentages of HepG2^shCtrl^ and Bel‐7402^shCtrl^ cells at G2/M phase were significantly increased to 70.61% and 67.10% with a obvious decrease in the percentages at G1 and S phase, suggesting that EPI might play its cytotoxic role in HCC cells by inducing G2/M phase arrest. However, in the HepG2^shMus81^ and Bel‐7402^shMus81^, cells underwent EPI treatment and the percentages at S phase were dramatically increased to 88.97 and 69.69% with a obvious decrease in the percentages at G1 phase, indicating that Mus81 knockdown induces a significant S‐phase arrest in HCC cells under EPI treatment.

### CHK1 is involved in S‐phase arrest induced by Mus81 knockdown

In view of the regulating role of CHK1 and CHK2 in S‐phase arrest, the small molecule inhibitor against CHK1 or CHK2 was used to determine whether these two kinases were involved in S‐phase arrest in Mus81‐depleted HCC cells. When HepG2^shMus81^ and Bel‐7402^shMus81^ cells was treated with CHK1 inhibitor and then EPI, the percentages of these two cells at S‐phase were significantly decreased to 28.14 and 21.84% with a obvious increase in percentages at G2/M phase to 65.60 and 69.10%, which were close to the levels of EPI‐treated HepG2^shCtrl^ or Bel‐7402^shCtrl^ cells (Fig. 4), suggesting an apparent involvement of CHK1 in S‐phase arrest of Mus81‐depleted HCC cells under EPI treatment. However, CHK2 inhibition only further improved the percentages at S phase of HepG2^shMus81^ and Bel‐7402^shMus81^ cells under EPI treatment to 92.30% and 74.96% with a decrease in percentages at G2/M phase to 1.97% and 22.93%, suggesting that CHK2 inhibition could aggravate S‐phase arrest, but not rescue it and CHK2, therefore, may not be the primary regulator in S‐phase arrest of Mus81‐suppressed HCC cell under EPI treatment.

**Figure 5:**
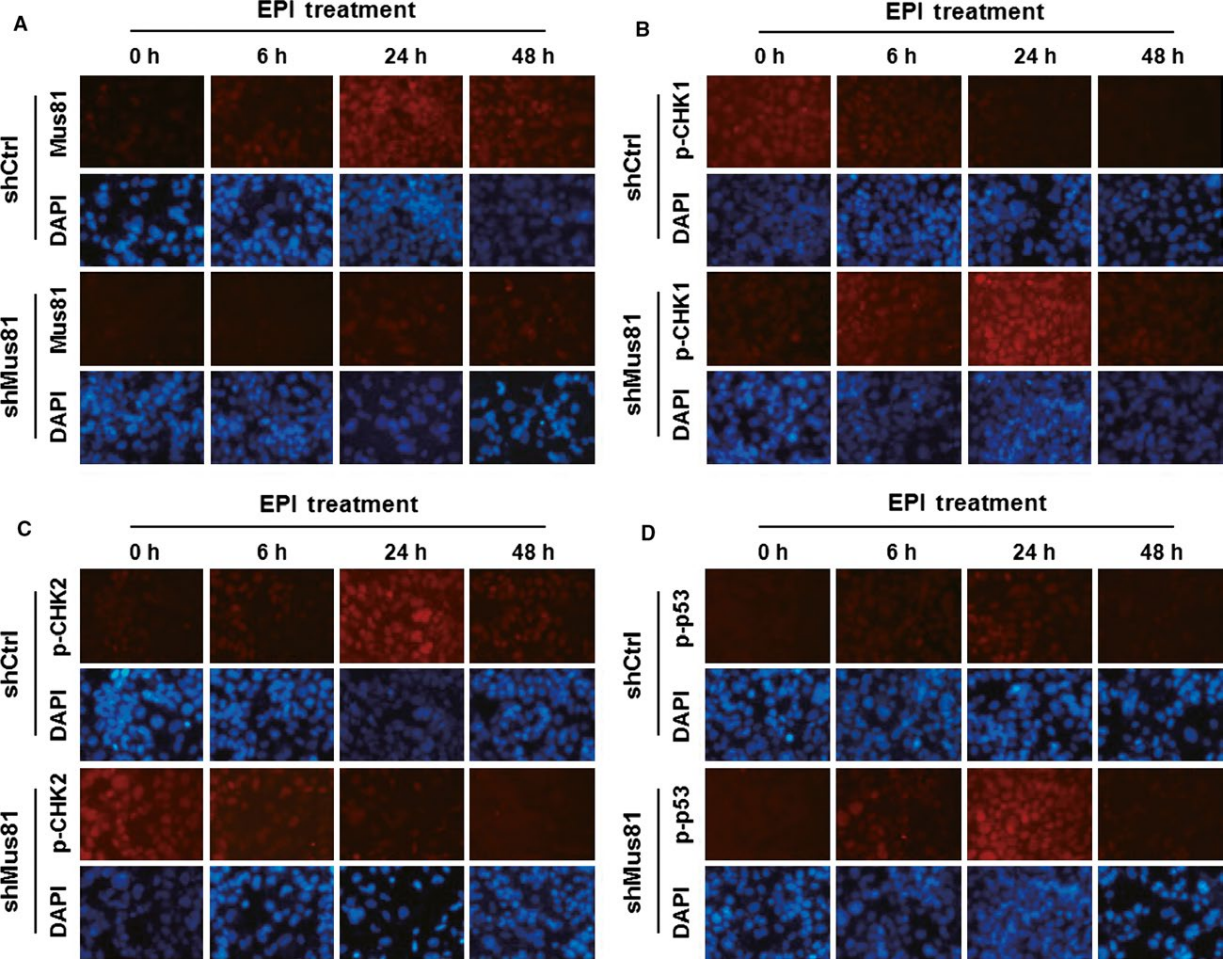
The activation of CHK1 pathway in Mus81 inhibited HepG2 cells under epirubicin (EPI) treatment. Time course for the expression of Mus81(A), phosphorylated CHK1 (B), phosphorylated CHK2 (C) and phosphorylated p53 (D) was determined by immunofluorescence assay in HepG2^shMus81^ and HepG2^shCtrl^ cells. Cell nucleus were counterstained with DAPI. Original magnification: (A–L, ×400).

Subsequently, an immunofluorescence assay was employed to further determine the dynamic change in the expression of Mus81, phosphorylated CHK1 (Ser317), and phosphorylated CHK2 (Thr68) in HepG2 cells under EPI treatment lasting 48h. The results showed that Mus81 expression was elevated in both HepG2^shCtrl^ and HepG2^shMus81^ cells, and reached its peak in 24 h after EPI treatment though Mus81 expression was significantly suppressed in HepG2^shMus81^ cells, suggesting that Mus81 expression is upregulated in response to EPI treatment. The expression of phosphorylated CHK1 (Ser317) in HepG2^shMus81^ cells also increased in 6 h after EPI treatment, which persisted up to 24 h, then slightly declined at 48 h. However, its expression maintained at a low level in HepG2^shCtrl^ cells after EPI treatment. Interestingly, the expression of phosphorylated CHK2 (Thr68) continued to decline in HepG2^shMus81^ cells after EPI treatment while significantly increased in HepG2^shCtrl^ cells under the EPI treatment and reached its peak in 24 h after treatment (Fig. 5). All these data demonstrated that CHK1 but not CHK2 is necessary to S‐phase arrest in Mus81‐inhibited HCC cells under EPI treatment.

### CHK1 pathway activation in Mus81‐suppressed HCC cells in vitro and in vivo under EPI treatment

**Figure 6:**
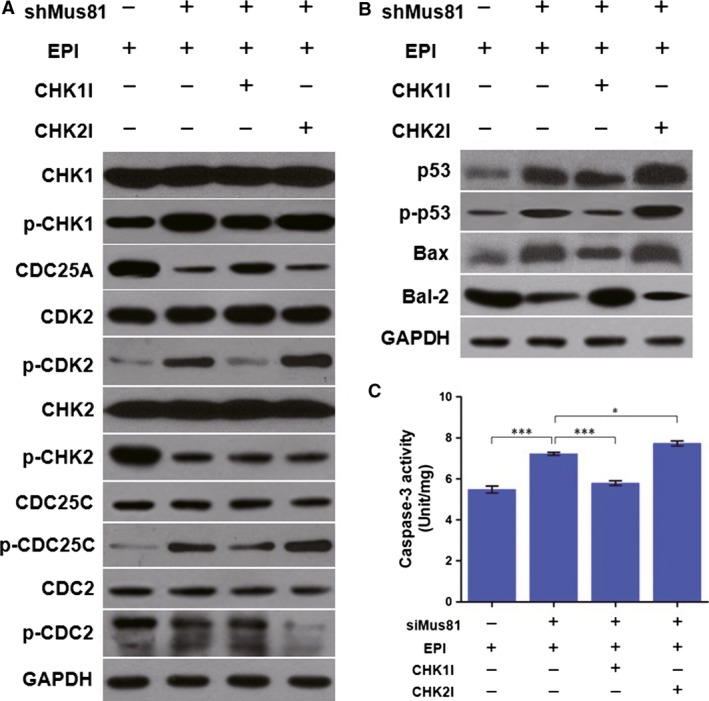
The activation of CHK1 and p53 pathway in Mus81‐depleted HepG2 cells. (A) The expression levels of CHK1 and CHK2 pathway were detected by western blot in HepG2 cells. (B) The expression of p53 pathway in HepG2 cells were also detected by western blot. (C) The activity of Caspase‐3 was also analyzed in HepG2 cells and the data were presented as mean ± SEM. p‐CHK1, phosphorylated CHK1 (Ser317); p‐CDK2, phosphorylated CDK2 (Tyr15); p‐CHK2, phosphorylated CHK2 (Thr68); p‐CDC25C, phosphorylated CDC25C (Ser216); p‐CDC2, phosphorylated CDC2 (Tyr15); p‐p53, phosphorylated p53 (Ser15); EPI: epirubicin; CHK1I, CHK1 inhibitor; CHK2I, CHK2 inhibitor. *P < 0.05; ***P < 0.001.

Western blot was employed to detect the expression of activated CHK1, CHK2, and their pathway in EPI‐treated HepG2^shCtrl^ or HepG2^shMus81^ cells. As shown in Figure 6A, the expression of phosphorylated CHK1 (Ser317) was elevated in HepG2^shMus81^ cells under the EPI treatment, but was suppressed by the treatment of CHK1 inhibitor. Interestingly, CHK2 inhibition could slightly improve the expression of activated CHK1 in EPI‐treated HepG2^shMus81^ cells. Meanwhile, the downregulation of CDC25A and the phosphorylation of CDK2 at Thr160 were all following the activation of CHK1, indicating the anticipation of CHK1 pathway in HepG2^shMus81^ cells under EPI treatment. On the other hand, the expression of phosphorylated CHK2 (Thr68) was high in EPI‐treated HepG2^shCtrl^ cells, but significantly decreased in HepG2^shMus81^ cells under EPI treatment or the sequential treatment of CHK2 inhibitor and EPI. However, CHK1 inhibition could slightly increase the expression of activated CHK2 in HepG2^shMus81^ cells under EPI treatment. In addition, the expression of phosphorylated CDC25C (Ser216) and CDC2 (Tyr15) was also suppressed in HepG2^shMus81^ cells under EPI treatment, especially in those cells treated with CHK2 inhibitor and EPI, and CHK1 inhibitor could slightly upregulate their expression levels, suggesting the inhibition of CHK2 pathway in HepG2^shMus81^ cells during the treatment of EPI. However, there were not obvious differences in expression levels of unphosphorylated CHK1, CDK2, CHK2, CDC25C, and CDC2 between HepG2^shMus81^ and HepG2^shCtrl^ cells whether with or without EPI treatment.

**Figure 7:**
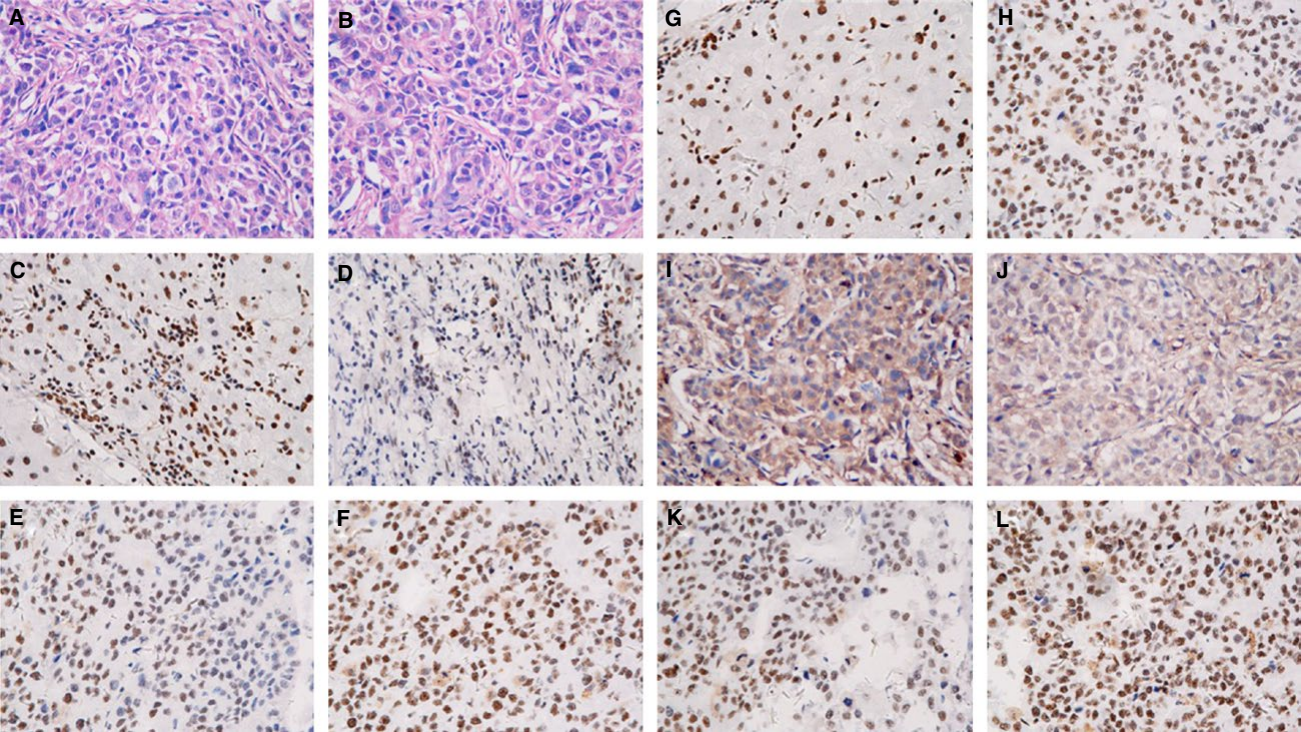
Expression of Mus81 and CHK1 pathway and the apoptosis levels in xenograft tumor generated from HepG2 cells with intraperitoneally epirubicin (EPI) injection. H&E staining for tumors generated from HepG2^shCtrl^ and HepG2^shMus81^ was showed in (A) and (B). The expression of Mus81, phosphorylated CHK1 (Ser317), CDC25A, and phosphorylated CDK2 (Tyr15) was detected by immunohistochemistry in HepG2^shCtrl^ (C, G, I, K) and HepG2^shMus81^ (D, H, J, L) tumors. Apoptosis was determined by terminal deoxynucleotidyl‐transferase‐mediated d‐UTP‐biotin nick end‐labeling (TUNEL) assay in HepG2^shCtrl^ (E) and HepG2^shMus81^ (F) tumors. Original magnification: (A–L, ×400).

In addition, IHC assay was applied to investigate the expression of CHK1 pathway in xenograft tumors under EPI treatment. As shown in Figure 7G–L, the upregulation of phosphorylated CHK1 and CDK2, and the downregulation of CDC25A were evidenced in tumors generated from HepG2^shMus81^ cells compared with those generated from HepG2^shCtrl^ cells, which was consistent with the results obtained from western blot and further verified the activation of CHK1 pathway in EPI‐treated HepG2^shMus81^ cells in vivo.

### Mus81 knockdown promotes EPI‐induced apoptosis of HCC cells in vitro and in vivo

**Figure 8:**
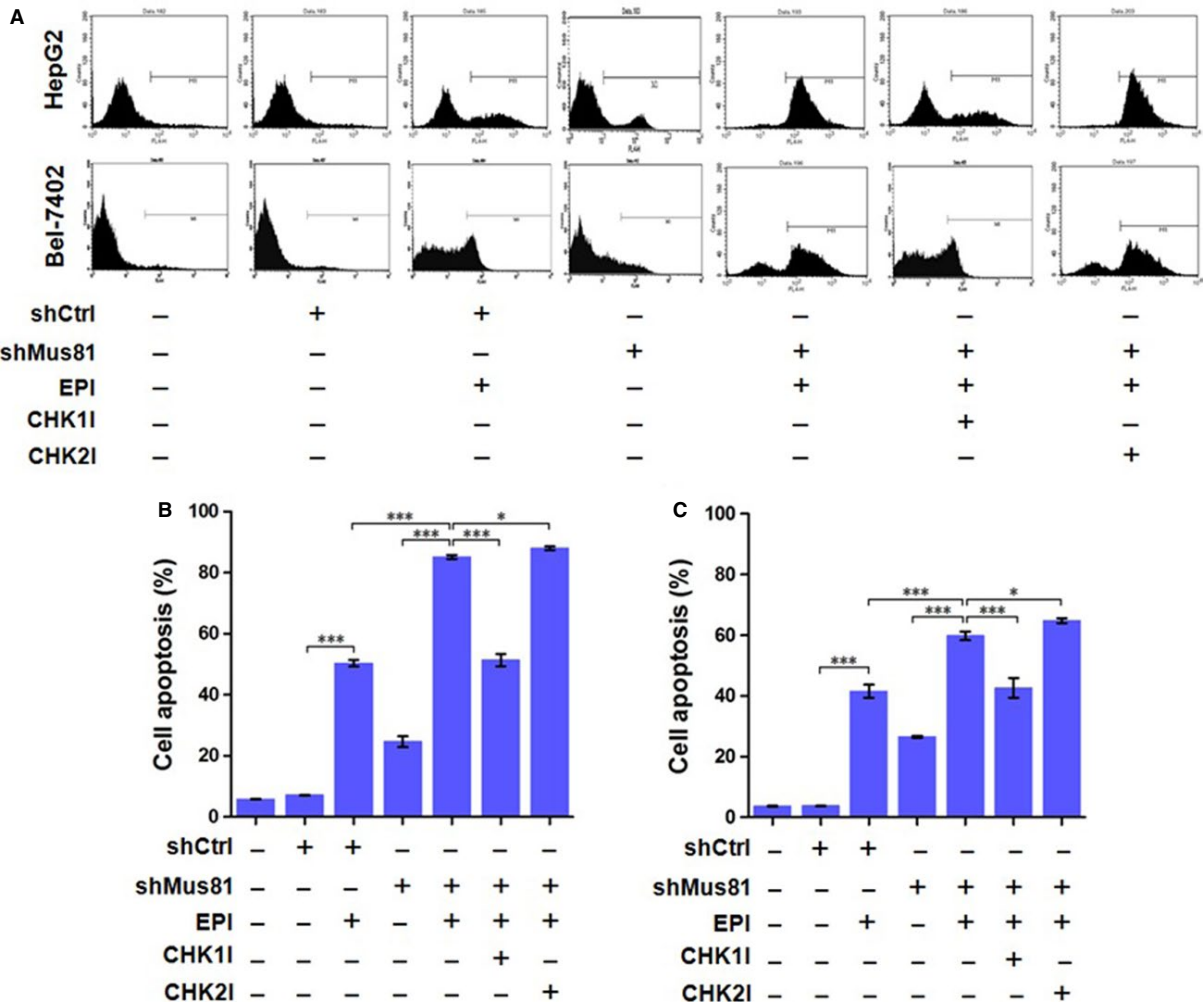
Mus81 knockdown affects apoptosis of human hepatocellular carcinoma HepG2 and Bel‐7402 cells under the treatment of epirubicin (EPI). (A) representative results of flow cytometric analysis. (B) Cell apoptosis analysis of HepG2 cells. (C) Cell apoptosis analysis of Bel‐7402 cells. And data are presented as mean ± SEM. CHK1I, CHK1 inhibitor; CHK2I, CHK2 inhibitor. *P < 0.05; ***P < 0.001.

For Mus81 knockdown caused a significant arrest of S phase in HCC cells, we further examined the apoptosis rate of HCC cells by flow cytometry analysis. As shown in Figure 8, the apoptosis rates of HepG2^shMus81^ and Bel‐7402^shMus81^ cells were slightly increased from 7.13 to 18.63% and from 3.76 to 12.53% when comparing with HepG2^shCtrl^ and Bel‐7402^shCtrl^ cells, suggesting Mus81 knockdown itself could slightly improve the apoptosis rate of HCC cells. After EPI treatment, the apoptosis rates of HepG2^shCtrl^ and Bel‐7402^shCtrl^ cells were significantly increased to 50.36 and 41.59%, and the apoptosis rates of HepG2^shMus81^ and Bel‐7402^shMus81^ cells were further increased to 85.17 and 59.78%, indicating that Mus81 knockdown could significant promote the cytotoxity of EPI to HCC cells. In addition, the TUNEL results in xenograft tumors showed that the apoptosis rate in tumors generated from HepG2^shMus81^ cells was significantly higher than those came from HepG2^shCtrl^ cells (Fig. 7E and F), having provided the in vivo proofs for the elevated apoptosis induced by EPI in Mus81 depleted HCC cells.

### Mus81 knockdown enhances EPI‐induced apoptosis in HCC cells through p53 and Caspase‐3 activation under CHK1 controlling

Since CHK1 played a critical role of S‐phase arrest in Mus81‐suppressed HCC cells under EPI treatment, we further explored its effects on the apoptosis of these cells. Our results showed that CHK1 inhibition could significantly decrease the apoptosis of EPI‐treated HepG2^shMus81^ and Bel‐7402^shMus81^ cells from 85.17 to 51.36% and from 59.78 to 42.64%, which were close to the apoptosis levels of EPI‐treated HepG2^shCtrl^ and Bel‐7402^shCtrl^ cells (Fig. 8). However, CHK2 inhibition slightly increased the apoptosis of EPI‐treated HepG2^shMus81^ and Bel‐7402^shMus81^ cells to 87.96 and 64.81%, suggesting the regulating role of CHK1 but not CHK2 in EPI‐induced apoptosis of Mus81‐suppressed HCC cells. For CHK1 often induces apoptosis through activating p53, we also observed the time course of the expression of phosphorylated p53 (Ser15) under EPI treatment. As shown in Figure 5, the expression of phosphorylated p53 was significantly increased in HepG2^shMus81^ and HepG2^shCtrl^ cells along with the lasting of EPI treatment and reached its peak in 24 h after EPI treatment, although its expression levels were obviously higher in HepG2^shMus81^ cells compared with HepG2^shCtrl^ cells, verifying the involvement of p53 in EPI‐induced apoptosis of HepG2^shMus81^ cells. To further analyze the interaction between CHK1 and p53, we detected the expression of phosphorylated p53 in EPI‐treated HepG2 cells by western blot. The results showed that the unphosphorylated and phosphorylated p53 were all increased in HepG2^shMus81^ cells compared with HepG2^shCtrl^ cells, but CHK1 inhibition could significantly suppress the expression of phosphorylated p53 (Fig. 6B), suggesting the activation of p53 was controlled by CHK1 in Mus81‐depleted HepG2 cells. Meanwhile, the expression of Bax was changed along with phosphorylated p53, but Bcl‐2 expression was going in the opposite direction. And CHK1 inhibition could suppress the expression of Bax but upregulate the expression of Bcl‐2 in EPI‐treated HepG2^shMus81^ cells. In addition, the activity of Caspase‐3 was also increased in HepG2^shMus81^ cells compared with HepG2^shCtrl^ cells under EPI treatment, but decreased by the treatment of CHK1 inhibitor, which was similar to the change trend of phosphorylated p53 expression in these cells and suggested the activation of p53/Bax/Caspase‐3 pathway under the controlling of CHK1. Moreover, CHK2 inhibitor could slightly increase the expression of phosphorylated p53 and Bax as well as the activity of Caspase‐3 in HepG2^shMus81^ cells, but decrease the expression of Bcl‐2 (Fig. 6B and C), which might be the mechanisms underlying the slightly increased apoptosis in HepG2^shMus81^ cells caused by CHK2 inhibition.

## Discussion

Although Mus81 is recently associated with the chemosensitivity in human malignancies such as colon cancer and breast cancer 16, 17, 18, its role in chemotherapy of HCC, a usually chemorefractory tumor 19, still remains unknown. In this study, our results showed that Mus81 knockdown by lentivirus‐mediated shRNA could remarkably improve the sensitivity of common chemotherapeutic drugs including EPI, 5‐FU, MMC, and cisplatin in human HCC HepG2 and Bel‐7402 cells, and the RI value of EPI was the highest among the four agents we tested. Moreover, the HCC chemotherapy mouse model had also shown an elevated sensitivity of EPI and 5‐FU in xenograft tumors came from Mus81‐depleted HepG2 cells without obvious weight loss of mice bearing these tumors. EPI, a doxorubicin analogue, is one of the most active single agents used in chemotherapy of HCC, but its application was limited because of the side effects including acute dose‐limiting hematological toxicity and cumulative dose‐related cardiac toxicity 20. Since Mus81 inhibition dramatically improves the sensitivity of EPI in HCC cells as well as animal xenograft model, the dose of EPI to achieve equivalent response could be significantly decreased in condition of Mus81 being suppressed, which is obviously helpful to diminish the dose‐dependent toxicity effects of EPI in HCC chemotherapy. 5‐FU is a widely used antitumor agent and still the mainstay of chemotherapy, also in HCC 21. It was recently documented that Mus81 inhibition by siRNA could improve the sensitivity of 5‐FU in human breast cancer MCF‐7 and T47D cells 17. Our results therefore provided in vitro as well as in vivo evidence in HCC to further confirm the regulation of Mus81 in sensitivity of 5‐FU. Though the sensitivity of MMC and cisplatin was also increased in Mus81 inhibited HCC cells, the RI values of these two agents were obviously lower than EPI and 5‐FU. However, the extent of the sensitivity of these two agents increased in Mus81‐disrupted HCT116 colon cancer cells was significantly higher than other agents such as methylmethane sulfonate (MMS) and hydroxyurea 16. This difference may be caused by different response of chemotherapeutic agents in different tumor cells, but the underlying mechanism is still worth demonstrating in the future. Whatever, all these results have provided in vitro as well as in vivo evidence, at first time, to show the ability of Mus81 knockdown to improve the chemosensitivity of HCC.

It was documented recently that Mus81 inhibition could induce a G2/M phase arrest in human breast cancer cells under the treatment of 5‐FU, suggesting that cell cycle arrest might contribute to enhanced sensitivity by Mus81 deficiency 17. Therefore, we analyzed the cell cycle distribution of EPI‐treated HCC cells, and found that Mus81‐depleted HepG2 and Bel‐7402 cells showed a obvious S‐phase arrest under the treatment of EPI, while the negative control cells showed a G2/M phase arrest at the same condition, which is the common response of HCC cells to EPI 20. CHK1 and CHK2 were proved to be essential for cell cycle arrest before mitosis in response to DNA damage 22, and these two kinases was all activated by Mus81 deficiency in HCT116 colon cancer cells 16. We therefore guess that CHK1 or/and CHK2 would be involved in the cell cycle arrest induced by Mus81 knockdown. To test this hypothesis, we treated HepG2 and Bel‐7402 cells with CHK1 or CHK2 inhibitor before EPI treatment and analyzed cell cycle distribution of these cells again. The results showed that CHK1 inhibitor could rescue the S‐phase arrest induced by Mus81 knockdown while CHK2 inhibitor slightly aggravated S‐phase arrest instead of rescuing it. Immunofluorescence assay also showed that EPI treatment increased the phosphorylation level of CHK1 in Mus81‐inhibited HepG2 cells at 6 h, which persisted up to 24 h, then slightly declined at 48 h. Oppositely, phosphorylated CHK2 was significantly decreased in Mus81‐inhibited HepG2 cells after EPI treatment while increased obviously in EPI‐treated negative control HepG2 cells. In addition, the results of western blot evidenced a significant increase in phosphorylated CHK1, but an obvious decrease in phosphorylated CHK2 in Mus81‐depleted HepG2 cells under EPI treatment. All these results indicated the critical regulating role of CHK1 in S‐phase arrest of Mus81‐depleted HCC cells while CHK2 might be pivotal in EPI‐induced G2/M phase arrest in those HCC cells with undisrupted Mus81.

We continued to investigate the detailed signaling pathway of CHK1 in S cell cycle arrest induced by Mus81 knockdown. Recent studies from various laboratories have shown that activated Chk1 phosphorylates the CDC25A and leads to its degradation, which prevents the activation (dephosphorylation at Tyr15) of CDK2 and halts the cells at S‐phase cell cycle 22, 23, 24. In this study, we indeed evidenced the decreased expression of CDC25A and the increased phosphorylation level of CDK2 in Mus81‐suppressed HepG2 cells under EPI treatment, and CHK1 inhibitor could reverse this change, implicating the involvement of CHK1/CDC25A/CDK2 pathway in S‐phase cell cycle induced by Mus81 knockdown. The IHC results also confirmed the activation of CHK1/CDC25A/CDK2 pathway in xenograft tumor generated from Mus81‐inhibited HepG2 cells under EPI treatment. Meanwhile, the activation of CDC25C (phosphorylation at Ser216) and CDC2 (dephosphorylation at Tyr15), the main downstream kinases of CHK2 in inducing G2/M phase arrest, was obviously decreased in Mus81‐suppressed HepG2 cells, but significantly increased in negative control cells after EPI treatment, which was in coincidence with the expression of phosphorylated CHK2. These findings suggested the regulation of CHK2/CDC25C/CDC2 pathway in EPI‐induced G2/M phase arrest in HCC cells with normal Mus81 expression, but further excluded the anticipation of this pathway in S‐phase arrest of Mus81‐suppressed HCC cells 22, 23. Get together, all these results demonstrated the main regulation role of CHK1 pathway in S‐phase arrest induced by Mus81 deficiency.

In advanced HCC, cancer cells usually become resistant to apoptosis 25, 26. Thus, investigating the effect of Mus81 knockdown on apoptosis of HCC cells is highly desirable. Our results showed that Mus81 knockdown could remarkably increase apoptosis of HepG2 and Bel‐7402 HCC cells by 69.12% (50.36 vs. 85.17%) and 43.74% (41.59 vs. 59.78%) under EPI treatment, respectively. TUNEL results from the HCC chemotherapy mouse model also showed an elevated apoptosis rate in tumors generated from Mus81‐inhibited HepG2 cells. These in vitro and in vivo proofs indicated that the promotion in apoptosis might be another mechanism underlying the enhanced chemosensitivity of Mus81‐depleted HCC cells. As the most important regulator in apoptosis, the activity of p53 was increased in Mus81‐dificient mouse embryonic fibroblasts (MEFs) in response to MMC and p53 inactivation could completely rescue the MMC hypersensitivity of Mus81‐depleted MEF cells, indicating the functional interplay of p53 and Mus81 though the molecular mechanism underlying this interplay was unknown 27. Mus81 inhibition also could increase apoptosis and p53 expression in MCF‐7 breast cancer cells after 5‐FU treatment 17. In this study, our results of immunofluorescence and western blot indeed evidenced an elevated expression of phosphorylated p53 in Mus81‐suppressed HepG2 cells after EPI treatment while CHK1 inhibitor could downregulate the activity of p53 to almost normal level. These results indicated that CHK1 is epistatic to p53 in EPI‐induced apoptosis of Mus81‐suppressed HCC cells, and therefore might be the mediator underlying the interplay of Mus81 and p53. In addition, the expression of Bax, a p53 target apoptotic protein, was changed along with p53 in Mus81‐depleted HepG2 cells, but Bcl‐2 expression changed in the opposite direction. Moreover, the activity of Caspase‐3, also an important apoptotic target gene of p53 28, 29, was found to be significantly increased in Mus81‐suppressed HepG2 cells under EPI treatment and this increase could be turned down by CHK1 inhibitor, suggesting a pivotal role of CHK1/p53/Bax/Caspase‐3 pathway in apoptosis of Mus81‐suppressed HCC cells in response to EPI 30.

Though our results mentioned above have firstly suggested an interaction between Mus81 and CHK1 in human HCC cells, a previous study has actually identified an interplay between Mus81 activity at stalled forks and the CHK1‐dependent DNA damage checkpoint during S phase when replication forks have collapsed in *Schizosaccharomyces pombe* 31. Recent studies also showed in human osteosarcoma cells that Mus81 is dependent in cell cycle defects and replication fork collapse caused by CHK1 inhibition and Mus81 depletion alleviates the S‐phase progression defects and decrease cell death induced by CHK1 deficiency 32, which to some extent agreeing with our results obtained in HCC cells that CHK inhibition could rescue the S‐phase arrest and apoptosis induced by Mus81 knockdown. Mre11 nuclease is even implicated to be the link between CHK1 pathway and Mus81 in the formation of double‐strand breaks (DSB) induced by CHK1 inhibition 33. Whatever, this study has provided the novel evidence to insight into the interaction between DNA repair gene such as Mus81 and checkpoint gene such as CHK1, though the underlying molecular mechanism should be further demonstrated in the future.

**Figure 9:**
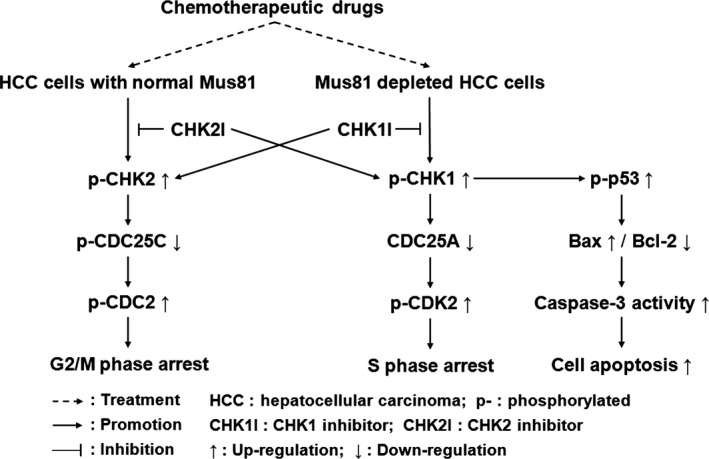
A schematic working model for presumed mechanisms underlying the effects of Mus81 knockdown on hepatocellular carcinoma (HCC) cells in response to chemotherapeutic drugs.

Interestingly, our results also showed that CHK2 inhibitor could slightly aggravating Mus81 deficiency‐induced S‐phase arrest and apoptosis in response to EPI. These data was consistent with the results from a recent study, which showing that dual inactivation of CHK2 and Mus81 leads to cell death of lymphoid cells despite the underlying mechanism remaining unknown 34. Our western blot results subsequently indicated that the activity of CHK1 pathway was slightly elevated in Mus81‐inhibited HepG2 cells after CHK2 inhibition, thereby promoting the activity of p53, Bax, and Caspase‐3, which might be the mechanisms underlying the deteriorative S‐phase arrest and apoptosis induced by CHK2 inhibition in Mus81 depleted HCC cells. On the other hand, our data also showed that CHK1 inhibition could slightly promote the activity of CHK2 pathway. These phenomena, we guess, may be the result from the functional complementary of CHK1 and CHK2 16, 23, 35, so inhibiting one of these two kinases could activate the other 36 (Fig. 9). From this perspective, we also could summarize the role of Mus81 knockdown as that Mus81 knockdown actually inhibits CHK2 by activating CHK1 and therefore converts G2/M arrest into S‐phase arrest 36 (Fig. 9). Although this presumption should be verified by further research, this finding has shed a light on combining CHK2 inhibitor with Mus81 knockdown in chemosensitization of HCC.

In conclusion, we demonstrated that Mus81 knockdown could significantly improve the chemosensitivity of HCC cells in vitro and in vivo by inducing S‐phase arrest and promoting apoptosis through CHK1 activation, which in turn activates CHK1/CDC25A/CDK2 pathway joined by CHK1/p53/Bax/Caspase‐3 branch. These data indicate Mus81 as a novel therapeutic target in chemosensitization of HCC, which will be helpful to develop a promising approach to improve the treatment of HCC.

## Conflict of Interest

None declared.
